# D-mannose reduces oxidative stress, inhibits inflammation, and increases treg cell proportions in mice with ulcerative colitis

**DOI:** 10.3389/fphar.2024.1454713

**Published:** 2024-11-01

**Authors:** Yuqing Lu, Yongjian Xiong, Shuangshuang Zhang, Boya Wang, Yuntao Feng, Zhuonan Pu, Kun Wei, Jun Chen, Dapeng Chen, Peng Zhang

**Affiliations:** ^1^ Department of Urology, The First Affiliated Hospital of Dalian Medical University, Dalian, China; ^2^ Comparative Medicine Department of Researching and Teaching, Dalian Medical University, Dalian, Liaoning, China; ^3^ Central Laboratory, First Affiliated Hospital of Dalian Medical University, Dalian, China; ^4^ Department of Thoracic Surgery, Shanghai Pulmonary Hospital, School of Medicine, Tongji University, Shanghai, China; ^5^ Beijing Neurosurgical Institute, Capital Medical University, Beijing, China

**Keywords:** regulatory T cells, oxidative stress, infalmmatory bowel disease, d-mannose, intestinal immunity

## Abstract

**Background:**

Regulatory T (Treg) cells is required to dampen immune responses against intestinal microbiota, which aid in a healthy body to promise that the resident gut microbiota should not attract the attention of the immune system. Inflammation and inflammatory bowel disease (IBD) can be induced if the immune system fails to ignore the resident gut microbiota and targets them instead. D-mannose, a common monosaccharide in nature, has been shown to ameliorate multiple autoimmune diseases. This study aimed to investigate the therapeutic effect of D-mannose on mice ulcerative colitis (UC) induced by 2,4,6-trinitrobenzene sulfonic acid (TNBS), and elucidate its underlying mechanisms.

**Methods:**

To simulate human IBD, we constructed a mouse model of UC by injecting TNBS into the colon.

**Results:**

Our results demonstrated that D-mannose treatment effectively alleviated TNBS-induced UC in mice, as evidenced by the amelioration of UC symptoms. D-mannose treatment significantly reduced inflammation by decreasing the expression of proinflammatory cytokines and inflammation mediators. D-mannose treatment also significantly inhibited oxidative stress, promoted the expression of GSH and SOD, decreased the expression of MDA. Mechanistically, D-mannose upregulated the proportion of both CD4(+) Tregs and CD8(+) Tregs.

**Conclusion:**

In summary, our study provides the first evidence of the therapeutic effect of D-mannose on mice with UC, which is likely mediated by upregulating Treg proportions.

## 1 Introduction

Inflammatory bowel disease (IBD) encompasses a group of chronic nonspecific inflammatory disorders, including Crohn’s disease (CD) and ulcerative colitis (UC) (Kaplan, 2015). The prevalence of IBD is high in the West, with rates exceeding 0.3% in Europe, North America, and Oceania. However, since 1990, the incidence of IBD has been increasing in Asia, Africa, and South America, and currently (Ng et al., 2017), approximately 3.9 million people worldwide suffer from IBD (Sudabeh Alatab et al., 2020), with the global burden of the disease on the rise. The symptoms of IBD, such as diarrhea, abdominal pain, rectal bleeding, and weight loss, significantly impact the patients’ quality of life (Wang et al., 2021). Additionally, IBD is often accompanied by refractory parenteral complications, including glomerulonephritis, anemia, osteoporosis, and Parkinson’s disease, among others (Camacho-Soto et al., 2018; Kim and Chang, 2014; Li et al., 2021; Ott and Schölmerich, 2013). Furthermore, persistent inflammation increases the risk of colorectal cancer (Nadeem et al., 2020; Stidham and Higgins, 2018). Therefore, further research is needed to explore the pathogenesis and treatment of IBD.

Treg cells are a special subgroup of T cells, and their main function is to suppress immune responds, thereby protecting the body from autoimmune diseases. Increasing evidence suggests that dysregulation of regulatory T (Treg) cells is involved in the pathogenesis of IBD (Chen and Sundrud, 2016; Zenewicz et al., 2009). A large quantity of Treg cells in the intestinal mucosa maintains tolerance to food and symbiotic bacteria, playing an essential role in the maintenance of the intestinal immune microenvironment (Sharma and Rudra, 2018). Treg cells manage the interactions between cells and the secretion of IL-10 to stabilize intestinal immunity (Plitas and Rudensky, 2016). Moreover, Treg cells suppress the differentiation and response of helper 17 T cells (Th17 cells), which are closely related to the pathological process of IBD (Geng and Xue, 2016; Ogino et al., 2011; Yamada et al., 2016). Dysregulation of these processes has been implicated in the pathogenesis of IBD in humans and mice (Britton et al., 2019). Based on the functional characteristics of Treg cell regulation, recent studies have revealed the potential of Treg cell therapy as an immunosuppressive strategy in IBD. Treg cell management has been shown to alleviate UC in different mouse models (Karlsson et al., 2011), and a Phase I/IIa clinical study of ovalbumin-specific Treg cells in 20 patients with refractory CD demonstrated good tolerability (Desreumaux et al., 2012). Furthermore, multiple drugs have been demonstrated to alleviate UC by regulating the differentiation and function of Treg cells (Liu H et al., 2020; Zhang et al., 2019), exhibiting a promising future for pharmacologic studies on Treg cells. CD4 (+) Treg cells are the earliest discovered and most extensively studied type of Treg cells. CD8 (+) Tregs play a key role in mucosal tolerance, with CD8 (+) CD28 (−) Treg cells preventing experimental colitis in mice and an increased proportion of CD8 (+) CD28 (−) Treg cells in the blood of colitis mice treated with mesalazine (Ceeraz et al., 2021; Ménager-Marcq et al., 2006).Traditional treatments for IBD mainly involve nonbiological therapies such as aminosalicylates, thiopurines, and steroids (Burger and Travis, 2011).

In recent years, the emergence of biological agents has also attracted considerable attention (Danese et al., 2015; Tong et al., 2021). However, many side effects and disease complications of these drugs have been reported, such as nausea, anorexia, decreased cells, and even an increased risk of infection and malignancy (Abraham et al., 2017; Andersen and Jess, 2014; Siegel et al., 2009). Due to the advantages of oral administration, minimal risk of antibody formation, and low production cost, the development of small molecule drugs is expected (Lucaciu et al., 2020). D-mannose is a heterotopic isomer of glucose at the C-2 position, which widely exists in Chinese medicinal herbs such as Dendrobium officinale, Huidouba, Grifola frondosa, and multiple fruits containing free mannose (Hu et al., 2016). D-mannose is absorbed and metabolized through the intestine and has no reported adverse effects on humans at present (Sharma et al., 2014). It has been shown to be beneficial in multiple fields, such as preventing acute urinary tract infections in women (Domenici et al., 2016; Kranjčec et al., 2014) and identifying insulin resistance at an early stage (Ferrannini et al., 2020). In recent years, the therapeutic potential of D-mannose in addressing autoimmune diseases has garnered escalating interest, bolstered by compelling evidence of its immunomodulatory capabilities across a spectrum of conditions, including autoimmune diabetes, osteoporosis, airway inflammation, and inflammation triggered by wounds, among others (Guo et al., 2018; Shi and Yin, 2017). In particular, the results of the current study have shown that D-mannose can promote the latent form of TGF-β activation and inducing naive CD4 (+) T cells to produce Treg cells, thereby ameliorating autoimmune diabetes and airway inflammation (Zhang et al., 2017). However, the effect of D-mannose on intestinal inflammation has not been reported.

This study aimed to investigate the anti-inflammatory activity of D-mannose on TNBS-induced UC in mice and evaluate and discuss the effects of D-mannose on CD4 (+) Treg cells and CD8 (+) Treg cells.

## 2 Materials and methods

### 2.1 Reagents

D-mannose (purity ≥98%) was purchased from Sigma–Aldrich (St. Louis, MO, United States), while Sulfasalazine (SASP) was acquired from Tianjin Kingyork Group Co. Ltd. (Tianjin, China). COX-2 and iNOS antibodies were procured from Cell Signaling (Boston, MA, United States). Red blood cell lysate was provided by Beijing Solarbio Science and Technology Co. Ltd. (Beijing, China). CD4, CD25, CD8, CD127, CD28, CD16/CD32 antibodies and cell membrane breaking lysate were provided by eBioscience. Unless otherwise stated, all chemicals utilized in this study were procured from Sigma-Aldrich (St. Louis, MO, United States).

### 2.2 Animals

Forty male C57BL/6 mice, aged between 6 and 8 weeks, were procured from the laboratory animal center (certificate: SYXK (Liao) 2018–0007) at Dalian Medical University (Dalian, China). The mice were acclimated to laboratory conditions (a temperature of 23°C, a 12-hour light/dark cycle, 50% humidity, and unrestricted access to food and water) for a period of 2 weeks prior to the commencement of the experiments. The experimental protocol, which was meticulously crafted to alleviate pain and discomfort for the animals, received approval from the Animal Experiment Ethics Committee of Dalian Medical University (approval number: AEE20046). Mice were individually housed and underwent a 12-h fasting period prior to the experiments.

### 2.3 Experimental design

The experiment was divided into 4 groups (n = 10/group). Group I was sham operation control group and was given ethanol in colon. Group II was the untreated UC group. Under the influence of ethyl carbamate anesthesia, a catheter was introduced via the anus to the level corresponding to the curvature of the spleen. Subsequently, consistent with the established protocol, 0.1 mL of TNBS dissolved in ethanol (50% v/v) was administered into the colon at a dosage of 125 mg/kg. Beginning one-hour post-procedure, the mice were granted unrestricted access to food and water. Group III constituted the model group, which received treatment with SASP (100 mg/kg body weight) administered intragastrically and dissolved in normal saline. SASP, a widely utilized anti-inflammatory medication in the clinical management of IBD and other ailments, was employed in this study as a positive control to evaluate the effects of D-mannose on UC. Group IV was the UC model group treated with D-mannose (1.1M, supplemented with D-mannose in drinking water). Previous studies provided clues about the optimal dose, so we chose to use that dose to validate the therapeutic effect of the drug (Liu Y J et al., 2020). Seven days later, blood samples were procured from the ocular region, and the mice were euthanized through cervical dislocation. Subsequently, tissue samples including the spleen, mesenteric lymph nodes, and colon were harvested from the deceased mice.

The condition of the mice, including fecal consistency, fecal bleeding, and weight loss, was observed and recorded daily, and the disease activity index was graded according to the criteria (Dong et al., 2021). The spleen and colon were dried and weighed to calculate the colon weight-length ratio and spleen index (SI) to assess the degree of inflammation in the UC (Wang et al., 2021) mouse model. The collected colon samples were used for a variety of biochemical analyses, including western blotting and hematoxylin and eosin (H&E) staining. The protein was extracted from 100 mg of colon tissue, followed by transferring to the membrane, blocking, applying antibody to the membrane, and finally developed using enhanced chemiluminescent reagents. Collected colon samples were first immobilized, followed by dewaxing and dehydration, H&E staining, and evaluated according to histological scoring criteria (Zhou et al., 2018). Serum was obtained after the collected blood sample was centrifuged at 1,000 g at 4°C for 10 min and stored at −20°C for subsequent ELISA experiments. The experiments followed established methods (Dong et al., 2021).

### 2.4 Oxidative stress analysis

0.1 g of colon tissue was weighed and placed into 1,000 mL of extracting solution, followed by centrifugation at 8000 g for 10 min at 4°C. to obtain colon tissue homogenate. The contents of GSH, MDA and SOD were detected by commercial kit (Beijing Solarbio Science and Technology Co., Ltd., Beijing, China) according to the instructions.

### 2.5 Flow cytometry

Fresh spleen and mesenteric lymph nodes were placed in 3 mL PBS buffer of 10% FBS and then ground at a low temperature for 2 minutes with a 5 mL syringe and a 200-mesh nylon mesh. The grind is then transferred to the 15 mL EP tube. Splenic tissue homogenates were centrifuged at 1100 RPM for 10 min, while heparin-treated peripheral blood was centrifuged at 2240 RPM for 5 min to allow cells to settle on the bottom. 2 mL of red blood cell lysate was added to the sample and left for 15 min in a dark environment. The initial treatment was then performed by centrifugation at 2240 RPM for 5 min. After a washing step of 10 mL of RPMI-1640 solution, we were able to obtain a pure single lymphocyte suspension. For mesenteric lymph nodes, centrifugation was performed at 2000 RPM for 5 min to locate the deposited cells at the bottom. After cleaning again with 10 mL of RPMI-1640 solution, purified single lymphocyte suspension was finally obtained.

The prepared single-cell suspension underwent secondary filtration through a 200-mesh nylon screen before being transferred into a 1.5 mL Ep tube. Thereafter, the cell concentration was adjusted to 1 × 10^6 cells/mL. Before staining, cells were pre-incubated with purified CD16/CD32 monoclonal antibodies at 4°C for 15 min. Equal amounts of 50 μL cell suspension were added to each tube. The antibodies CD4, CD8, CD25, CD28, and CD127 were mixed in an appropriate volume of flow cytometry staining solution and added to the cells, resulting in a final staining volume of 100 μL (i.e., 50 μL cell sample and 50 μL antibody mixture). Gently vortex mixed with a pulse, incubated in the dark at 4°C for over 30 min. Cells were washed with flow cytometry staining solution, 2 mL per tube, centrifuged at 600 g for 5 min at room temperature. The supernatant was discarded. The cells were then resuspended in 250 μL of flow cytometry staining solution and analyzed by flow cytometry.

### 2.6 Statistical analysis

The animal experiments and data analysis were conducted according to a single-blind study design. For data that follows a normal distribution, one-way ANOVA was used to compare data among three or more groups, and Student's t-test was used for statistical analysis between two groups of data. For data that does not follow a normal distribution, the non-parametric Kruskal–Wallis test is employed. All experiments were repeated at least three times, and a P-value of less than 0.05 was considered statistically significant. All statistical analyses were conducted using GraphPad Prism 8.3.0.

## 3 Results

### 3.1 Toxicology of D-mannose *in vivo*

**FIGURE 1:**
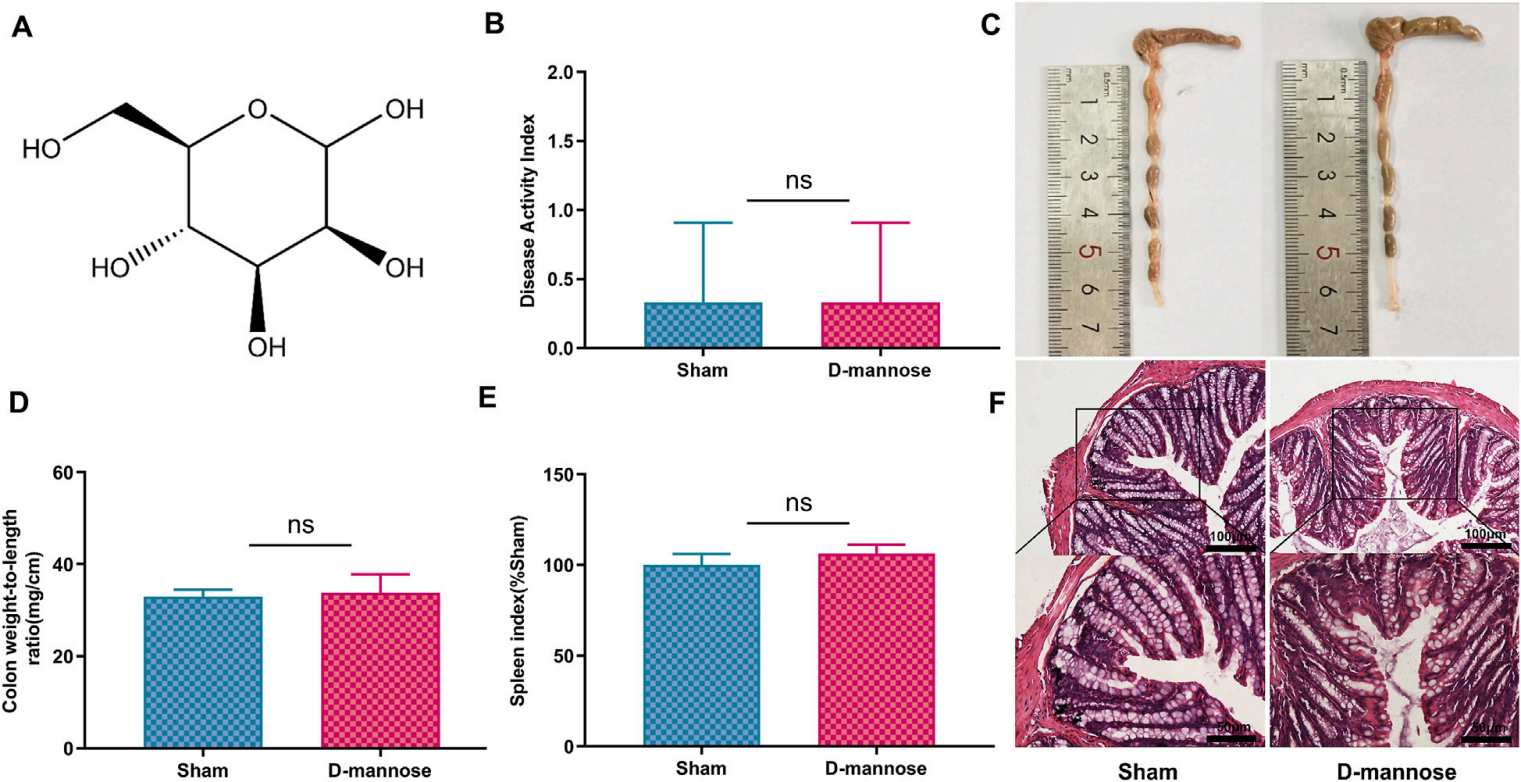
Toxicology of D-mannose *in vivo*. **(A)** Chemical structure of D-mannose; **(B)** Disease activity index; **(C)** Colon length; **(D)** Weight to length ratio of colon; **(E)** Spleen index; **(F)** Hematoxylin and eosin staining in each group. Scale: upper panel is 100 μm, lower panel is 50 μm.

In the study, we administered D-mannose to experimental mice through their drinking water for a continuous period of 7 days, and conducted a series of physiological and histological assessments. The results indicated that the use of D-mannose did not exert any significant impact on the DAI of the mice, the colon weight-to-length ratio, the SI, or the colon length (Figures 1B–E). Furthermore, histological examination of the colon tissue via H&E staining also demonstrated that there were no notable differences in colon tissue structure between the sham-operated group and the mice treated with D-mannose (Figure 1F). Collectively, these findings suggest that D-mannose did not exhibit significant *in vivo* toxicity over the course of its 7-day continuous use.

### 3.2 D-mannose improved symptoms of TNBS-induced UC

**FIGURE 2:**
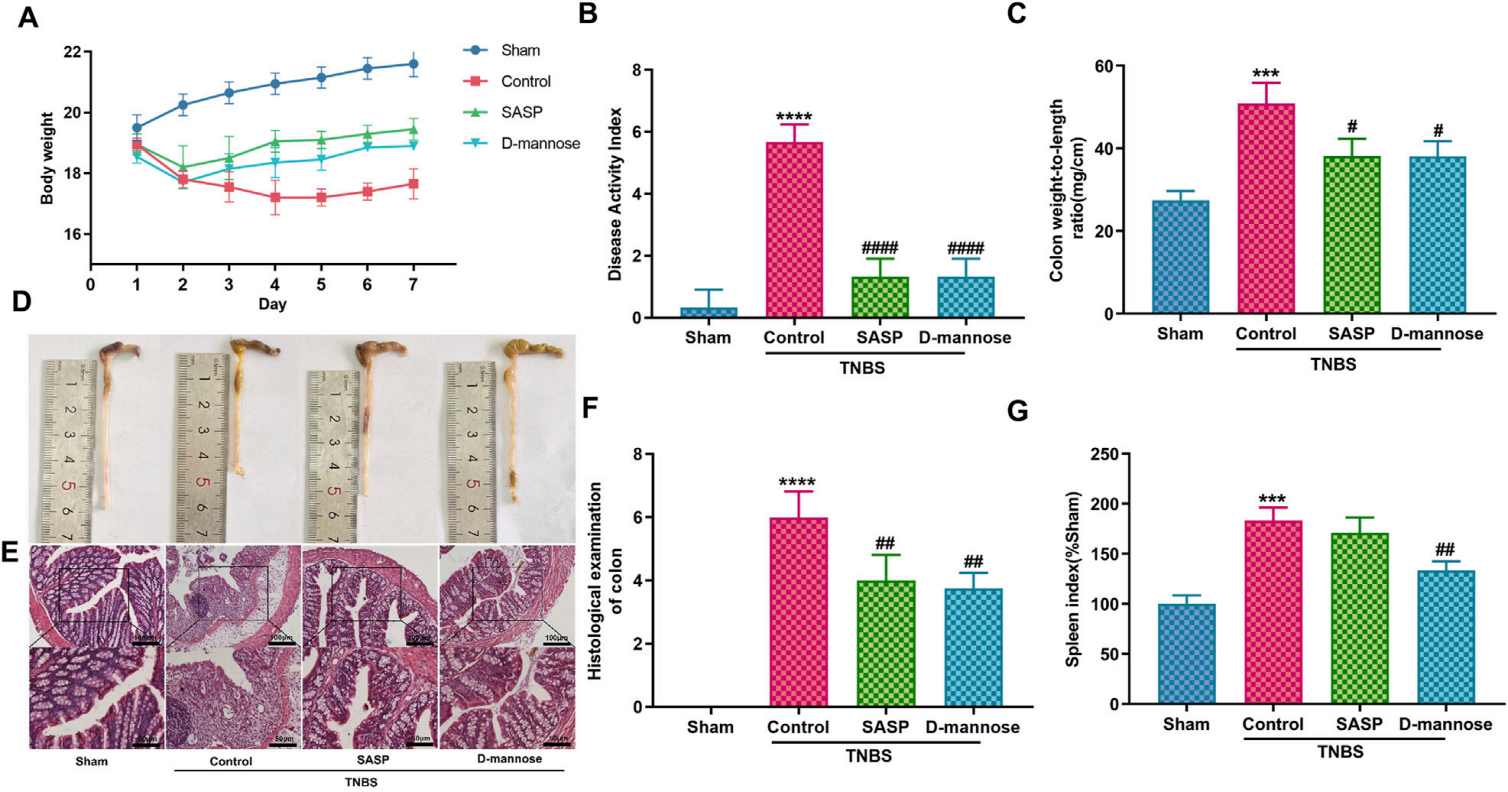
Effect of D-mannose on ulcerative colitis (UC) symptoms in mice. **(A)** Body weight; **(B)** Disease activity index; **(C)** Weight to length ratio of colon; **(D)** Colon length; **(E, F)** Hematoxylin and eosin staining in each group, and corresponding scores (Scale: upper panel is 100 μm, lower panel is 50 μm); **(G)** Spleen index. Data were expressed as mean ± SD. ***p < 0.001 compared with sham group; ****p < 0.0001 compared with sham group; #*p* < 0.05 compared with TNBS model control group. ##*p* < 0.01 compared with TNBS model control group. #### *p* < 0.0001 compared with TNBS model control group. n = 10 mice.

Table 1 presents details of defecation in mice. TNBS-induced UC mice exhibited increased stool frequency, irregular yellow stools, and occasional discharge of red fluid from the anus, accompanied by abdominal masses, lethargy, and loss of appetite. These symptoms persisted for approximately 4 days, with some improvement after 3 days. In contrast, mice treated with D-mannose or SASP showed symptom improvement on day five. The untreated model group exhibited significant weight loss, short colon length, high colon weight-to-length ratio, high DAI scores, and high SI, indicating successful replication of the UC model using TNBS. However, mice administered D-mannose exhibited a notable increase in body weight and colon length, accompanied by a reduction in DAI scores, colon weight-to-length ratio, and SI when compared to the untreated model group. These findings suggest that D-mannose may attenuate the macroscopic tissue damage associated with TNBS-induced UC (Figure 2).

**TABLE 1:** The symptoms of each group of mice were recorded.

Group	Symptoms	Symptom duration
Sham	No obvious symptoms	/
TNBS model control	Unformed stool, defecate frequency increases, anus outflow red or dark red liquid, defecate occult blood test positive, lack of appetite	Symptoms peaked on day 3 and persisted for about 7 days
SASP	Unformed stool, defecate frequency increases, anus outflow red or dark red liquid, defecate occult blood test positive, lack of appetite	Symptoms lasted for about 4 days
D-mannose	Unformed stool, defecate frequency increases, anus outflow red or dark red liquid, defecate occult blood test positive, lack of appetite	Symptoms lasted for about 4 days

Mice with TNBS-induced UC exhibited colonic pathological damage, such as severe thickening of the mucosal muscular layer, epithelial damage, infiltration of inflammatory cells into the lamina propria, and destruction of glandular structures (Figure 2E). Treatment with SASP or D-mannose improved epithelial integrity, reduced inflammatory cell infiltration, and mitigated mucosal muscular layer thickening. The pathological scores were markedly lower in both the D-mannose and SASP groups when contrasted with the TNBS group. Conversely, the TNBS group exhibited significantly elevated scores in comparison to the sham group (Figure 2F). These findings suggest that D-mannose alleviates the pathological changes in the colon caused by TNBS and mitigates the progression of experimental UC.

### 3.3 Effects of D-mannose on inflammation and oxidative stress

**FIGURE 3:**
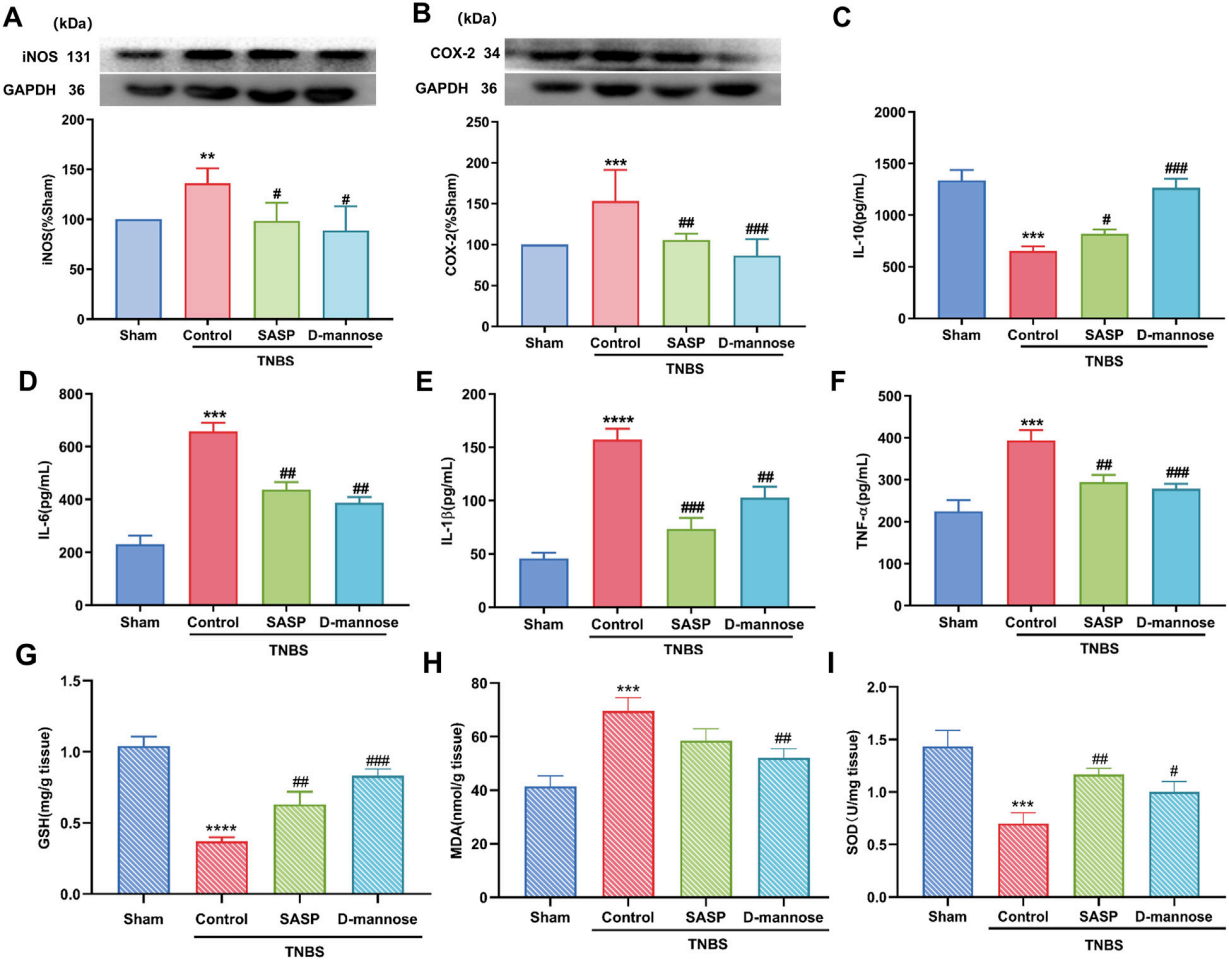
Effect of D-mannose on inflammatory factors and oxidative stress. Western blotting analysis of the secretion of inflammation-related proteins: **(A)** iNOS; **(B)** COX-2; ELISA analysis of inflammatory cytokines; **(C)** IL-10; **(D)** IL-6; **(E)** IL-1β; **(F)** TNF-α; Oxidative stress index analysis; **(G)** GSH; **(H)** MDA; **(I)** SOD. Data were expressed as mean ± SD. **p < 0.01 compared with sham group. ***p < 0.001 compared with sham group; ****p < 0.0001 compared with sham group; #*p* < 0.05 compared with TNBS model control group. ##*p* < 0.01 compared with TNBS model control group. ###*p* < 0.001 compared with TNBS model control group. n = 10 mice.

TNBS-induced UC upregulated inflammation-related proteins. We have detected an increase in the expression levels of iNOS and COX-2 proteins in the model group mice. In comparison, the expression levels of these two proteins are significantly lower in the sham-operated group mice (Figures 3A, B). ELISA results showed that IL-10 was significantly decreased in TNBS-UC compared to the sham group, while IL-6, IL-1β, and TNF-α were significantly increased (Figures 3C–F). In contrast, D-mannose significantly ameliorated these alterations when compared to the untreated TNBS model control group, thereby suggesting that D-mannose curtails the expression of pro-inflammatory factors while augmenting the expression of anti-inflammatory factors.

In addition, we analyzed the indexes related to oxidative stress. The results indicated that, relative to the Sham group, the model group demonstrated a significant decline in GSH content and SOD activity. Following treatment with D-mannose, the decrease of GSH content and SOD activity and the increase of MDA level in the model group were reversed (Figures 3G–I). These results suggest that D-mannose can ameliorate intestinal damage in IBD by inhibiting TNBS-induced oxidative stress and inflammatory response.

### 3.4 Effect of D-mannose on proportion of CD4 (+) treg cells

**FIGURE 4:**
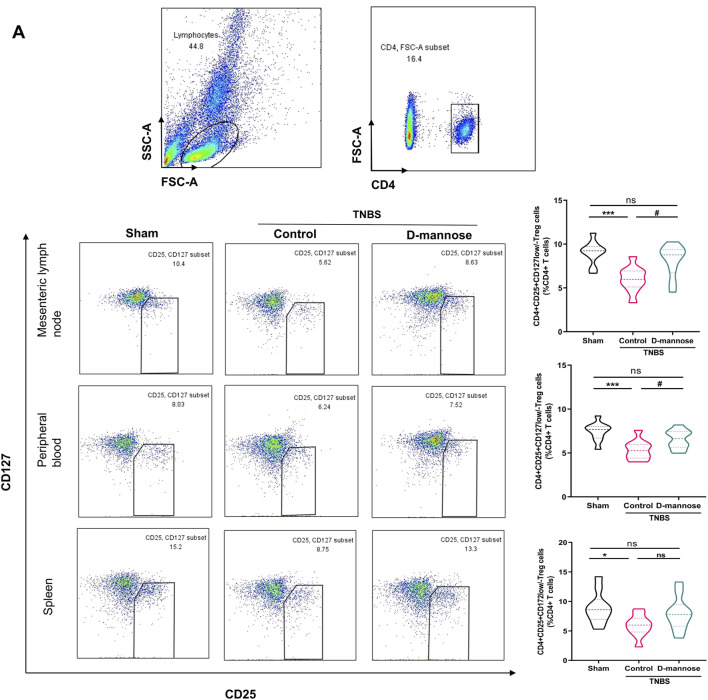
Effect of D-mannose on CD4 (+) Treg cells. **(A)** Flow cytometric sorting of CD4 (+) CD25 (+) CD127 (low/-) Treg cells in mesenteric lymph nodes, peripheral blood and spleens. Data were expressed as mean ± SD. *p < 0.05 compared with sham group. ***p < 0.001 compared with sham group; #*p* < 0.05 compared with TNBS model control group.

We evaluated the proportions of CD4 (+) CD25 (+) CD127 (low/-) Treg cells in mesenteric lymph nodes, peripheral blood, and the spleen. Notably, compared to the sham-operated group, the TNBS-induced group exhibited significantly reduced percentages of CD4 (+) Treg cells. In contrast, the groups administered D-mannose demonstrated an opposite trend, with increased Treg cell percentages (Figure 4A). The Treg cell percentages in mice treated with D-mannose were significantly higher than those in the TNBS-induced group, indicating that D-mannose may stimulate Treg cell differentiation in intestinal immunity. IL-10 secretion reflects Treg cell function, and it was significantly reduced in the TNBS group compared to the sham group. In contrast, both SASP and D-mannose groups showed increased IL-10 secretion, particularly in mice treated with D-mannose (Figure 3C). These findings suggest that D-mannose activates the expression of Foxp3 and IL-10, and reverses the dysregulation of CD4 (+) Treg cells, providing a potential therapeutic target for D-mannose treatment.

### 3.5 Effect of D-mannose on proportion of CD8 (+) treg cells

**FIGURE 5:**
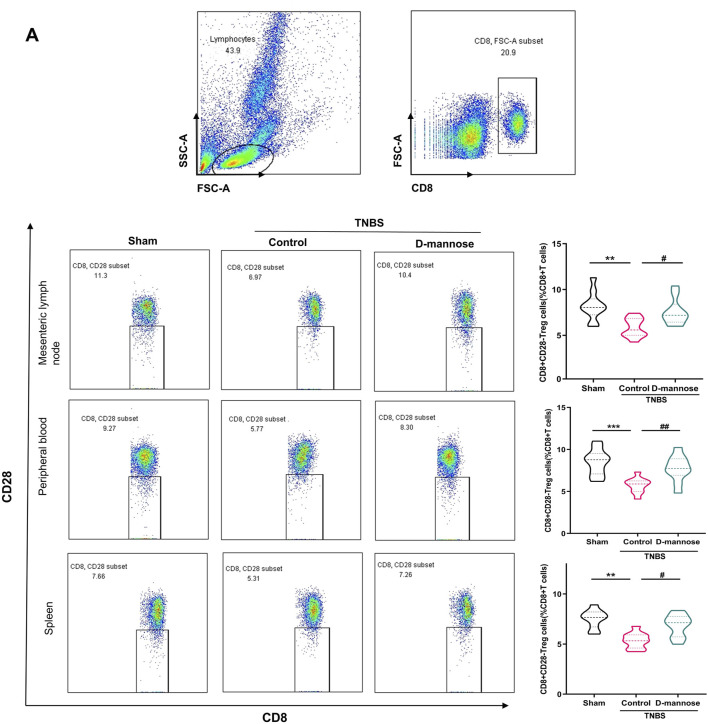
Effect of D-mannose on CD8 (+) Treg cells in peripheral blood. **(A)** Flow cytometric sorting of CD8 (+) CD28 (−) Treg cells in mesenteric lymph nodes, peripheral blood and spleens. Data were expressed as mean ± SD. **p < 0.01 compared with sham group; ***p < 0.001 compared with sham group; #*p* < 0.05 compared with TNBS model control group; ##*p* < 0.01 compared with TNBS model control group. n = 10 mice.

We analyzed the proportions of CD8 (+) CD28 (−) Treg cells in mesenteric lymph nodes, peripheral blood, and the spleen (Figure 5). The TNBS-UC group exhibited a significantly reduced percentage of CD8 (+) Treg cells. Conversely, the groups treated with D-mannose demonstrated an opposite trend. Mice administered D-mannose exhibited significantly higher percentages of CD8 (+) Treg cells compared to the untreated model group, indicating that D-mannose possesses the ability to increase the proportion of CD8 (+) Treg cells (Figure 5).

## 4 Discussion

In this study, D-mannose treatment relieved TNBS induced UC symptoms and improved the inflammatory response in mice by inhibiting the release of pro-inflammatory factors, inhibiting oxidative stress, and upregulating the proportion of Treg cells.

The TNBS-induced UC model is extensively employed to investigate the pathogenesis of IBD and to evaluate potential therapeutic agents (Antoniou et al., 2016). Mice treated with TNBS exhibited serious UC symptoms, and their spleens were significantly enlarged, which is considered as compensatory enhancement of immune function. Flow cytometry results showed that mice treated with TNBS presented a significantly decreased proportion of Treg cells in mesenteric lymph nodes, peripheral blood, and spleen, suggesting that systemic and intestinal immune disorders are key factors in the formation and development of UC. Treatment with D-mannose substantially reversed this change, indicating its potential to modulate the immune response.

During the acute phase, recruitment and infiltration of inflammatory cells result in the copious secretion of pro-inflammatory factors, including TNF-α, IL-6, and IL-1β, which induce the release of various inflammatory mediators and damage to enterocytes (Kany et al., 2019). We demonstrated that D-mannose treatment ameliorated the progression of intestinal pathology in UC mice. D-mannose significantly curtailed the secretion of pro-inflammatory cytokines and suppressed the expression of inflammatory mediators, thereby alleviating inflammation. The balance between oxidative stress factors and antioxidant system entities is important for the pathogenesis of IBD. In our study, we observed that the MDA level was significantly diminished in the D-mannose treatment group when compared to the TNBS group, while the antioxidant activities of SOD and the content of GSH were markedly augmented.

In this study, D-mannose upregulated the proportion of Treg cells and reversed their dysfunction to maintain immune homeostasis in mice with TNBS-induced UC. Research indicates that D-mannose can enhance the immunosuppressive function of CD4 (+) Treg cells by increasing the production of IL-10 to reduce the release of pro-inflammatory factors (including IL-1β, IL-6, and TNF-α, etc.), thereby inhibiting T-cell-mediated immune response, and ultimately alleviate intestinal inflammation (Ko and Auyeung, 2014; Owczarczyk-Saczonek et al., 2018). CD8 (+) CD28 (−) Treg cells have been demonstrated to have beneficial effects in IBD and other autoimmune diseases, although their molecular mechanisms remain to be explored (Ceeraz et al., 2021). This dual regulatory effect aids in restoring the integrity and function of the intestinal mucosa, diminishing the pathological progression of IBD. Moreover, D-mannose may further modulate the function of Treg cells by influencing the composition of the gut microbiota (Zhang et al., 2021). The gut microbiota plays a crucial role in the pathogenesis of IBD by directly affecting the differentiation and function of Treg cells through the production of short-chain fatty acids (such as butyrate) and other metabolites (Ramanan et al., 2023; Su et al., 2022). D-mannose could indirectly influence the homeostasis of Treg cells by altering the metabolic activity of the gut microbiota, offering a multifaceted intervention strategy for the treatment of IBD. In summary, D-mannose provides a novel perspective for the treatment of IBD by upregulating the proportions of CD4 (+) Treg and CD8 (+) Treg cells and restoring their functions. This therapeutic approach not only directly targets the suppression of inflammatory responses but also involves the restoration of immune homeostasis and the modulation of the gut microbiota, offering potential for comprehensive treatment of IBD.

Our study is subject to certain limitations. The mechanism by which D-mannose regulates Treg cells in the intestine is not uncovered in this study. The relationship between D-mannose and Treg were described by multiple studies mainly in three aspects. (1) D-mannose promotes the generation of integrin αvβ8 and ROS to activate the TGF-β pathway (Zhang et al., 2017). (2) Mannose regulates the PPARγ pathway that is critical to the differentiation and maturation of Treg cells (Xu et al., 2015). (3) Mannose alters the intestinal microbiome (Sharma et al., 2018). Bile acids produced via intestinal commensal bacteria metabolism, as well as short-chain fatty acids, such as butyric acid, regulate innate immunity and Th17/Treg homeostasis (Campbell et al., 2020; Haase et al., 2018; Hang et al., 2019; Hang et al., 2020; Yamashiro, 2017). These experimental results indicate the complicated regulatory network of D-mannose on Treg cell regulation, providing beneficial implications for the future study of the mechanism of D-mannose in regulating Treg cells in the intestine.

In conclusion, D-mannose can alleviate the symptoms of UC. The therapeutic effect of D-mannose is achieved through the upregulation of the proportion of Treg cells and the inhibition of oxidative stress, which ultimately results in the reduction of pro-inflammatory cytokines and inflammatory mediators. Considering the mechanisms of action of D-mannose and its lack of significant side effects in clinical applications, it has the potential to become a promising drug for IBD patients seeking effective and safe treatment options. To comprehensively assess the efficacy and safety of D-mannose in the treatment of IBD, further research and clinical trials are essential. These studies will help determine the optimal dosage, duration of treatment, and its effectiveness across different subtypes of IBD, thereby providing patients with more precise and personalized treatment choices.

## Data Availability

The original contributions presented in the study are included in the article/Supplementary Material, further inquiries can be directed to the corresponding authors.
