# Supplementary material for: D-mannose reduces oxidative stress, inhibits inflammation, and increases treg cell proportions in mice with ulcerative colitis
Source: Front Pharmacol. 2024 Nov 1;15:1454713. doi: 10.3389/fphar.2024.1454713 (PMC11563948; doi:10.3389/fphar.2024.1454713)

Legends: N=sham control; M=TNBS-induced UC model; S=SASP treated group; D= D-mannose treated group.

1. **Figure 3-panel A: WB analysis of iNOS protein (left: iNOS; right: GAPDH)**

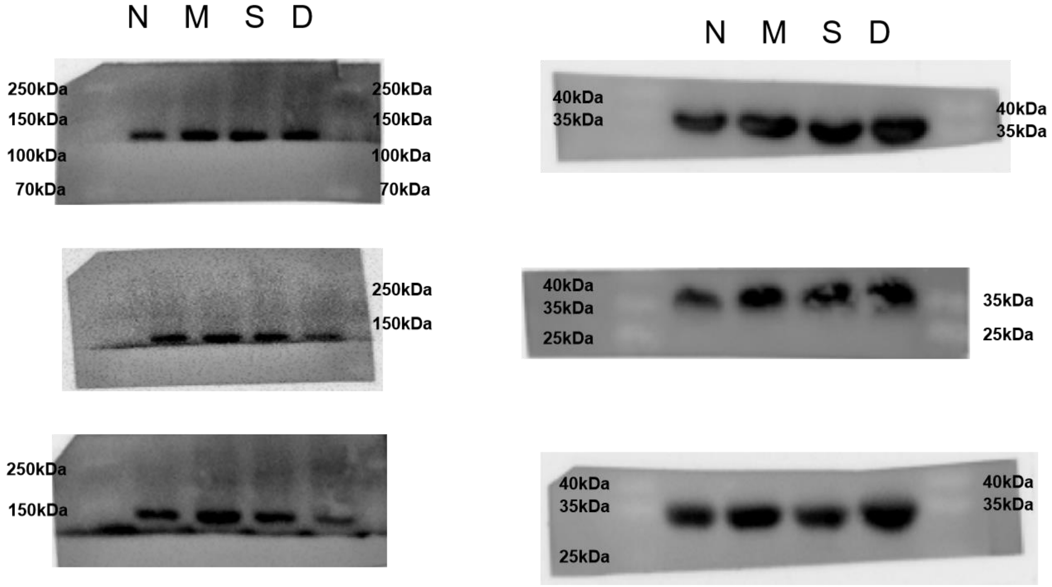

2. **Figure 3-panel B: WB analysis of COX-2 protein (left: COX -2; right: GAPDH)**

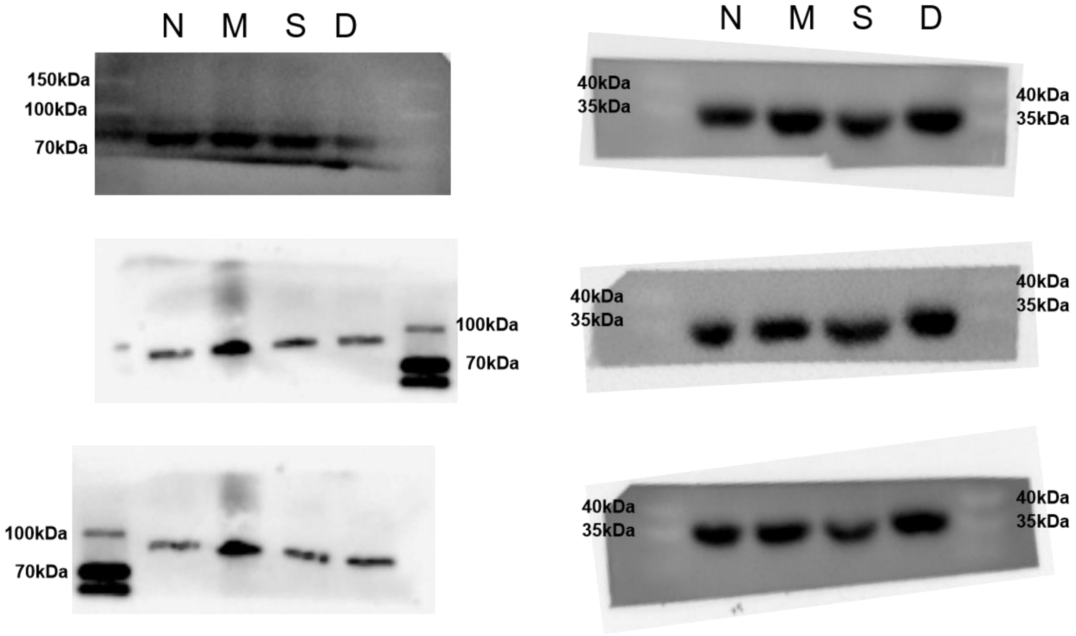

Supplement: Supplementary file 1 [file DataSheet1.pdf]
